# Visualizing Data Interoperability for Food Systems Sustainability Research—From Spider Webs to Neural Networks

**DOI:** 10.1016/j.cdnut.2023.102006

**Published:** 2023-09-29

**Authors:** Emily M. Jennings-Dobbs, Shavawn M. Forester, Adam Drewnowski

**Affiliations:** 1 Nutrient Institute, Reno, NV, United States; 2 Center for Public Health Nutrition, University of Washington, Seattle, WA, United States

**Keywords:** interoperability, ontologies, USDA FoodData Central, agriculture, climate change, food prices, nutrition, population health

## Abstract

Food systems represent all elements and activities needed to feed the growing global population. Research on sustainable food systems is transdisciplinary, relying on the interconnected domains of health, nutrition, economics, society, and environment. The current lack of interoperability across databases poses a challenge to advancing research on food systems transformation. Crosswalks among largely siloed data on climate change, soils, agricultural practices, nutrient composition of foods, food processing, prices, dietary intakes, and population health are not fully developed. Starting with US Department of Agriculture FoodData Central, we assessed the interoperability of databases from multiple disciplines by identifying existing crosswalks and corresponding visualizations. Our visual demonstration serves as proof of concept, identifying databases in need of expansion, integration, and harmonization for use by researchers, policymakers, and the private sector. Interoperability is the key: ontologies and well-defined crosswalks are necessary to connect siloed data, transcend organizational barriers, and draw pathways from agriculture to nutrition and health.

## Introduction

Transforming current food systems to become more sustainable while maintaining and improving the nutritional status of the population has become a global public health priority for international organizations, funders, academics, and policymakers [[1], [2], [3]]. Because food systems activities cover a vast range of activities (including agricultural production, aggregation, processing, distribution, retail, food purchase, consumption, disposal, and waste [4]), any such transformations will be extremely complex.

Ongoing diet sustainability analyses [5] have pointed to interactions and tradeoffs among the 4 food systems domains of nutrition and health, economics, society, and the environment. Diet sustainability analyses employ metrics and measures from multiple domains that are context specific and operate across space and time [5,6]. The goal of such analyses is to identify dietary patterns that respect planetary boundaries while assuring affordable food and nutrition security for all [4]. However, linking data sets from multiple domains can be challenging, and there is a scarcity of tools to integrate disparate data sets into a common analytical framework [5].

Formulating evidence-based policies for food systems transformation requires a harmonized data framework spanning the range from agricultural production to food processing, distribution, and retail, and then to food consumption patterns, nutrition recommendations, and health [4]. However, as others have noted [5], there are large gaps in food systems data, and not all data—public or private—are readily accessible. Notably, data on food prices are not always available [7], nutrient composition data may lack key nutrients [8], and data on the environmental impact of food production can differ across geographic regions and are subject to change as agricultural practices evolve with time. Being able to link agricultural production with nutrition is key, especially because the existing data on agricultural practices, soil quality, and livestock management are separate and distinct from data on dietary intakes, food composition, nutritional recommendations, and health. Data on the environmental impacts of food production in relation to nutrient density are necessary to feed the rising global population and prevent malnutrition without causing environmental destruction. Studies on sustainable, healthy diets would benefit from greater interoperability, defined here as expanding, integrating, and harmonizing the existing data into a common modeling framework.

Recent studies have summarized some key sources of available food systems data and have described possible methods and tools for their integration [5,9]. They found that linking agricultural, economic, and environmental data with nutrition and health databases was the major challenge because the data were either collected by different agencies, provided to potential users in different formats, and/or stored at different locations.

### Using USDA data as an interoperability example

The USDA provides leadership on food, agriculture, natural resources, rural development, and nutrition. Furthermore, it serves as a repository of data from multiple food systems domains ranging from agricultural production and nutrition economics to consumer behavior and public health outcomes [5,8]. Creating interoperability among USDA data has been identified as a worthwhile initiative. For instance, the Agricultural Research Data Network tool being created by the Agricultural Model Intercomparison and Improvement Project is intended to make historical USDA crop systems data accessible and interoperable [10].

To illustrate the challenges facing that project, the existing interoperability among USDA databases may be visualized in a spider web diagram (presented by John Finley at Nutrition 2020 Live; Supplemental Figure 1 [11]). This diagram helps identify areas in need of transformation by showing connections among USDA databases within the 4 sustainability domains. This web displays a vision for the future that coincides with USDA's efforts to advance food composition data infrastructure through the inclusion of a standardized ontology.

The goal of this study was to enhance the current spider web visualization by expanding the data sources, identifying crosswalks, and proposing a neural network-type visual harmonization of food data interoperability. Additionally, we address technical challenges that limit the interoperability of data and suggest food ontologies (e.g., LanguaL, FoodEx2, and FoodOn) that can aid in the integration of food systems data by providing a common classification of foods. The visualizations of food data connections and accompanying index of data sources can be used as data discovery tools and can help identify gaps in data interoperability required for food systems research.

### Brief Overview and Inclusion of Global Databases

**FIGURE 1:**
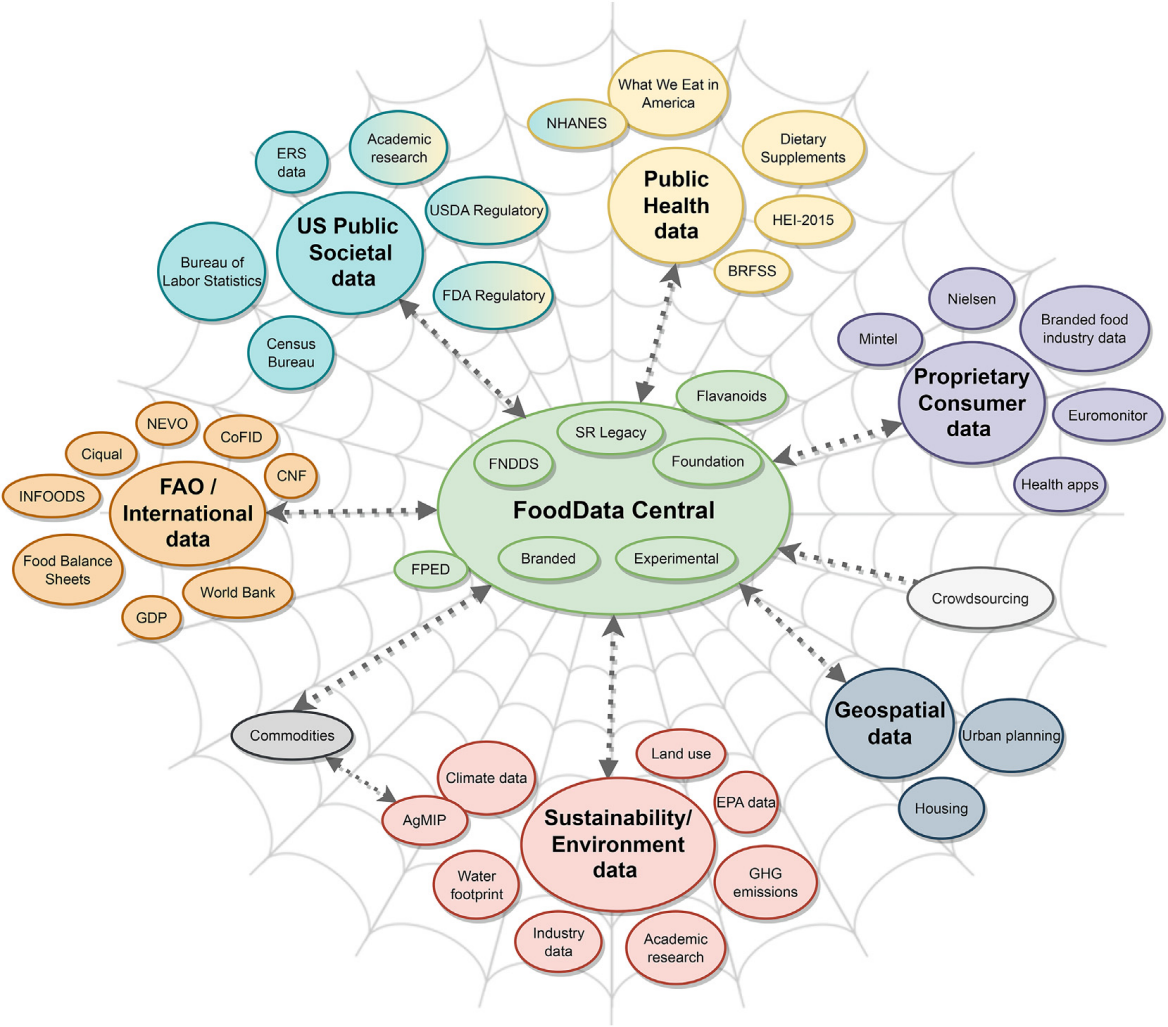
Expansion of food systems data sources to include global databases. This diagram is a general overview. Using FoodData Central as a starting point [12], arrows indicate connections to different types of data needed for food systems research. This type of mapping visualization provides an overview of data sources grouped by category, all of which are important components in food data systems, rather than identifying specific connections between data sources. The color indicates groups of data that are siloed together. Groups are not intended to be all encompassing, and data sources within each group often overlap. Abbreviations: EPA, Environmental Protection Agency; ERS, Economic Research Service; FDA, United States Food and Drug Administration; FNDDS, Food and Nutrient Database for Dietary Studies; FPED, Food Patterns Equivalents Database; GDP, Gross Domestic Product; GHG, greenhouse gas; HEI, Healthy Eating Index.

Figure 1 shows an initial revision of the USDA spider web (see Supplemental Figure 1) to represent a more global perspective [[11], [12]]. FAO International Network of Food Data Systems (INFOODS) is the leading source of multiple global food composition databases [13]. Otherwise, the majority of food composition data sources are region-specific (e.g., FAO/INFOODS Food Composition Table for Western Africa) or were developed by national public health agencies in Canada (Canadian Nutrient File), France (Ciqual), the Netherlands (NEVO) or elsewhere. Many high-income countries conduct nationally representative surveys of dietary patterns (National Diet and Nutrition Survey, INCA3, and Encuesta Nacional de Salud y Nutrición) but most lower- and middle-income countries do not. In the latter cases, food balance data serve as proxies for consumption.

The development of linkages between diet composition and its environmental impact has shown progress [5,14]. Researchers have manually linked foods and commodities with environmental impact estimates. Examples of this include dataFIELD [15], which facilitates linkages between NHANES and greenhouse gas emissions, and Poore and Nemecek’s [16] Multi-Indicator Global Database, which consolidates data on environmental impacts in relation to food production systems.

In general, LifeCycle Analyses of the environmental impact of food production have been calculated on a kilogram basis. Data have been collected on agricultural growing conditions including air quality, soil, irrigation, energy demand, and climate, as well as the amount of produce grown and when it was harvested. Collected geospatial data characterize crop coverage and location, food availability, loss and waste rates, and region-specific federal food systems. Other important environmental data include biodiversity loss, climate change, land use, and water use [6]. However, the kilogram is not a measure of nutrient density: that is, agricultural, geospatial, and environmental data do not provide nutritional data in terms of nutrients or bioactive components. Therefore, the environmental impact of food ought to be weighed against the nutritional value it provides. Such calculations will require a better integration of environmental data with food composition, dietary guidance, and health data.

Societal and geospatial data offer a new approach to health equity and population food preferences and eating habits. Geographic Information Systems data provide measures of community food access, choice, and preferences. Studies have shown that eating habits, diet quality metrics, and health outcomes vary predictably with area-level measures of socioeconomic status [17]. Societal data provide information that identifies characteristics of the population and is often used to better understand gender equity issues as well as the contribution of foods and food systems to social identity, community values, tradition, and culture [18]. Societal factors such as occupation, culture, and education can have direct impacts on food behaviors and overall health [19]. The movement toward sustainable healthy diets relies on community support and interaction.

### Expanding and visualizing the range of transdisciplinary data sources

The present visual demonstration is intended to serve as proof of concept. To gauge the current state of established connections between data in the 4 sustainability domains (health and nutrition, economics, environment, and society), data sources were identified from the USDA data catalog [20], and a nonsystematic review of current publications. Collected data sources were defined as either primary sources, which include foundational data such as food composition tables (e.g., FoodData Central); and secondary sources, which transformed or built on primary data to assess food or diet quality or provide diet-related information to consumers. A total of 200 data sources were recorded and categorized (Table 1). Some data sources were coded into multiple domains, for example, diet and health surveys were assigned to the categories of “health/nutrition” and “society.” A comprehensive index of data sources is provided in Supplemental Table 1.

**TABLE 1:** Categories of food-related data sources

Data source category	Description	No. of sources	Sustainability domain(s)
Primary data sources			
Food composition	Nutritional composition of foods and food products	97	Health/nutrition
Dietary Supplements	Composition and product labeling of dietary supplements	3	Health/nutrition
Commodities	Conversion of food products to agricultural commodities	2	Economics, environment
Dietary intakes	Dietary and health data collected from surveys	28	Health/nutrition, society
Prices and expenditures	Prices related to the production and sale of food products	11	Economics
Environmental	Greenhouse gas emissions, land use, water use	12	Environment
Geospatial	Locations of production, purchase, or availability of foods	7	Environment, society
Food balance Data	Food supply data by country by year (FAOSTAT)	2	Environment
Secondary applications			
Research applications	Tools for collecting and assessing food frequency questionnaires	14	Health/nutrition
Consumer applications	Computer applications (including mobile applications) for assessment of dietary intake by the general public	6	Health/nutrition
Dietary guidelines	Guidelines for recommended consumption of food and nutrients	8	Health/nutrition, society
Diet quality metrics	Measures of compliance with dietary guidelines	2	Health/nutrition
Nutrient profiling	Ranking foods based on their nutrient composition	3	Health/nutrition
Data characterization			
Food ontologies	Ontologies created for the harmonization of food-related data	5	Health/nutrition

Collected data sources organized into common categories.

We used the concept of interoperability as the common unifying theme. Based on standard public health definitions, the 3 pillars of interoperability are semantic interoperability, technical interoperability, and functional interoperability [21,22]. Semantic interoperability refers to the expansion of metadata or some information about the data, such as how it was collected. Technical interoperability involves using a common coding framework, such as an ontology or crosswalk to integrate multiple data sources. Finally, functional interoperability refers to translating or harmonizing the data so that they may be understood by researchers and the general public. The goal of this study was to illustrate technical interoperability.

### Data crosswalks

A crosswalk denotes the connection of 2 data sources through a common set of identifiers or indices, which can be used to link, merge, or join the data [23,24]. To better understand the nature of crosswalks, we assessed each data source for a documented relationship with other data sources in our demonstration sample. We developed a systematic approach to identify crosswalks. For each data source, we examined data documentation, codebooks, and supplementary descriptions, and then recorded crosswalks to applicable data sources directly identified by the publisher. In essence, a crosswalk documents and represents the relationship between elements from different data sources.

### Food ontologies

Ontologies create a common definition of a single term, consolidating understanding of terminologies within a domain and providing an organizational structure that can be used across data sources. Connecting multiple data sets to a common ontology serves to integrate the data sources even if there is no direct connection. Thus, an ontology is the most effective way to organize data that allows for technical interoperability—leading toward the harmonization of data within any domain.

LanguaL is the most commonly used ontology for food composition data and is already prevalent within USDA databases. However, LanguaL is limited by its scope: it was designed for implementation in food composition databases. Thus, the USDA has expressed interest in FoodOn and other ontologies and standards (FoodEx2, GS1, AGROVOC, and GACS), aiming to create a systematic ontologic framework that connects across nutrition, agriculture, sustainability, and public health data domains starting with FoodData Central [[25], [26], [27], [28]]. A single ontology linking the sustainability domains would be highly valuable and would provide a strong foundation for transdisciplinary research. Ontologies from multiple domains can be consolidated into a larger ontology to promote the exchange and harmonization of information across domains. For instance, FoodOn is an ontology that consolidates food composition terminology from LanguaL with terminology and structural relationships from Open Biological and Biomedical Ontology Foundry ontologies, such as the Compositional Dietary Nutrition Ontology and NCBITaxon.

### Visualizing food data systems

Research in the nutritional sciences may not utilize the full extent of data visualization, which is a valuable tool to transform raw data into something usable and understandable that can provide insight beyond the scope of traditional descriptive statistics. We need visualizations to communicate data findings in a way that is intuitive to the human brain so that we can identify patterns and trends. A variety of different visualizations of the same data may be used to highlight different insights.

**FIGURE 2:**
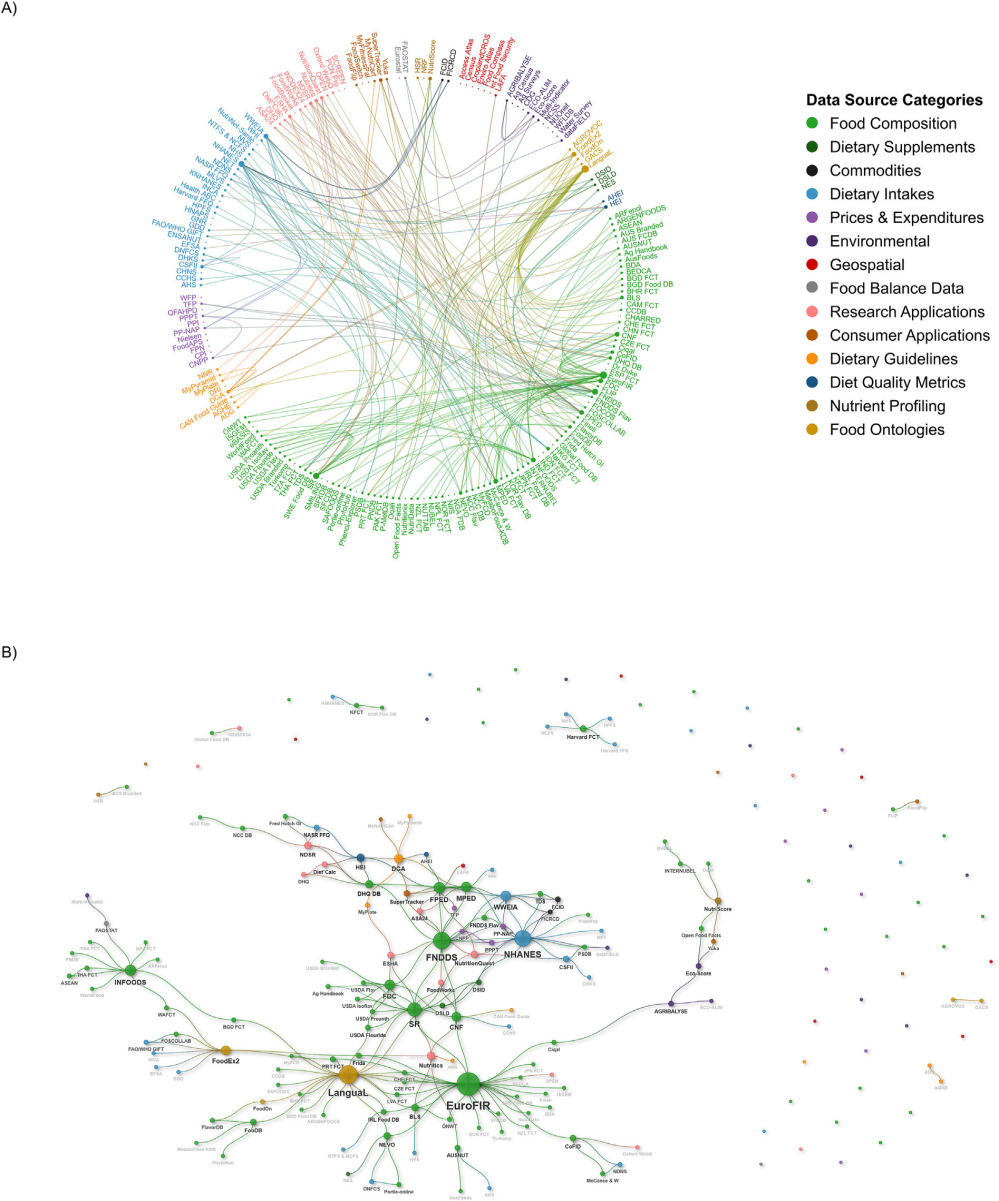
Food systems connectedness. Connections between food systems data are illustrated as (A) a chord diagram and (B) a neural network–type visualization. Each small circle represents one data source and lines connecting data sources represent crosswalks. The size of each data source circle reflects the number of crosswalks to the data source, and color indicates the data source category. A full index of databases including abbreviations and citations can be found in Supplemental Table 1.

At a base level, domain connectedness is often visualized using a chord diagram, which showcases one-to-one connections between data sources and allows for rapid identification of surface-level connections through the use of colors and density. Figure 2A illustrates the connectedness of food systems data sources identified in this study. Food composition data sources (light green circles) span >50% of the circle. Crosswalks to food composition data tend to fall into 3 clusters: USDA data sources (Food and Nutrient Database for Dietary Studies [FNDDS], National Nutrient Database for Standard Reference [SR], Food Patterns Equivalents Database [FPED], MyPyramid Equivalents Database, and others); EuroFir; and, to a lesser extent, INFOODS. EuroFir and INFOODS are both hubs of multiple food composition data sources. As a result, they have many crosswalks to other food composition sources but very few crosswalks to non–food composition sources.

There are strong connections between the food composition data sources and the food ontologies (yellow circles), with LanguaL being the most common food ontology. We also see strong connections between food composition and dietary intake data (blue circles). Except NHANES and What We Eat in America, which connect to a larger network of USDA sources, each dietary intake data source only has crosswalks to 1 or 2 other sources—each survey uses a different food composition data source, making reliable comparisons extremely difficult.

The key conclusions that can be drawn from this chord diagram are based on the absence of connections. Minimal connections are present for geospatial data (dark red, top right), environmental data (dark purple, upper right), and, to a lesser extent, food prices data (purple, bottom left). Our identification of these major data gaps confirms previous findings [5,9].

Although the chord diagram (Figure 2A) displays one-to-one connections of food systems data organized by domain, the neural network diagram (Figure 2B) reorients the same data as clusters of connected data sources, maintains color identification of domains, and allows visualization of secondary connections.

Some clusters are easy to interpret: both EuroFir and INFOODS are surrounded by clusters of food composition data sources. Other clusters are more complicated, such as the dense bundle of connections between various USDA data sources (Figure 2B, upper left). In this case, instead of having one data source that acts as a hub connecting to a series of isolated data sources, crosswalks are dispersed across multiple interconnected hubs (such as FNDDS, SR, NHANES, and others). Although these crosswalks allow for interoperability between many of the USDA data sources, they can be difficult to navigate.

This network diagram also reveals secondary connections. For example, at this time there is not a complete link between SR and FoodOn, but SR connects to LanguaL, which in turn connects to FoodOn. These secondary connections can serve as bridges that enable the integration of previously divided data sources, which is the goal of ontologies.

In its current state, food systems data are difficult to navigate. The power of the neural network visualization is its ability to highlight disconnectedness, yet be used to strategize impactful next steps in harmonizing food systems data. For example, food cost data are siloed, but economics is the greatest barrier to implementing healthful dietary actions. A first step in solving this problem would be to directly connect cost data sources with LanguaL, which would provide connections between cost and all food composition data connected to LanguaL. If a single ontology were implemented for all food systems domains, its visualization would resemble a family tree—one overarching ontology branching out to several domain-specific ontologies, which then branch out to the individual data sources.

### Limitations

Interoperability is necessary for advancing our understanding of food systems; however, not all data are of the same quality and intended for the same purposes. In this analysis, the validity and reliability of data sources were not assessed. Caution should be taken before analysis to ensure that high-quality data are prioritized.

Furthermore, the 200 data sources collected for this analysis do not comprehensively represent all data associated with food systems activities. Additional ontologies for food components (e.g., the Compositional Dietary Nutrition Ontology), chemical compounds (e.g., ChEBI), organisms (e.g., NCBITaxon – an automatic translation of the NCBI taxonomy database into obo/owl), and more may apply to food systems research.

### Technical challenges

Many technical challenges limit the interoperability of food systems data. Addressing these challenges is vital for the improvement of food systems data research.

### The need for comprehensive and accurate metadata

Technical interoperability through crosswalks and ontologies relies on semantic interoperability, which is the creation and expansion of extensive metadata. Metadata is the descriptive information that allows us to fully understand the data; it is a prerequisite for the successful joining of data sets. The first step of integration is to identify commonality between 2 data sets based on the descriptive metadata provided. However, we found that descriptive variables such as food type, nutrient name, food categories, analytical measures, and age of data varied across databases, thus preventing successful matches. Data users need to be able to match these descriptive variables, or they will be forced to subjectively create new corresponding descriptors to match the data. Ambiguous descriptive factors can lead to serious miscategorizations. For example, the Food Compass categorized 77% of all foods in the FNDDS 2015–2016 as ultraprocessed, including "carrots, boiled" [29].

Differing variable names also pose a challenge. For example, FNDDS, What We Eat in America and NHANES are deeply intertwined, yet the variable that is used to join these data sources has inconsistent names. It appears as “food_code” or “Food code” in FNDDS [30] but as “FDCD” or “USDA food code” in NHANES [31].

There are potential solutions. Most notably, in 2012, FAO/INFOODS established and implemented a standard for metadata requirements and many subsequent food composition data sets conform to those guidelines [32]. The FAO/INFOODS standard serves as a guide for good practices outside of the FAO as well. For example, the Food Composition Table for Bangladesh has implemented the FAO metadata documentation standard [33].

Additionally, the FAIR Guiding Principles for Scientific Data Management and stewardship aim to ensure the findability, accessibility, interoperability, and reusability of data [34]. The FAIR principles are built on the backbone of clear and comprehensive metadata, allowing for maximal interoperability between data sources.

### Inconsistent file formatting

Inconsistent file formatting is a major barrier to data integration [10]. Seamless integration occurs when all data follows a common format, such as Microsoft Excel or comma-separated values (CSV). Data in those formats can be easily read, integrated, and analyzed by the preferred integration coding languages, such as R, Python, and SQL. When the preferred file formats are not provided, transforming the data is a possibility, but the process requires specialty knowledge and can be expensive and time intensive.

Many international and regional nutrient composition databases are only available as scanned images or in pdf format (e.g., Indian Food Composition Tables, Latin America food composition tables). Those need to be converted to a more compatible format, such as CSV or Microsoft Excel, before being joined with other data. We have also identified data sources that are provided in a format not compatible with newer technologies. For instance, The Food Commodity Intake Database [35] is available as a CD-ROM. However, to improve the utility of these data, the Environmental Protection Agency has made these data available in CSV format and as an interactive tool through a collaboration with the University of Maryland and the Joint Institute for Food Safety and Applied Nutrition [36]. Conversely, recent file types with more complex structures, such as JSON and XML, require special knowledge, whereas SAS and STATA require proprietary software. Both situations make it difficult to restructure data into an interoperable format.

Encouragingly, however, we identified some data sources that have implemented systems for exporting data in multiple formats. For example, FooDB allows all data to be exported in CSV, XML, JSON, and MySQL dump file formats [37]. This versatility permits users to choose the file format that works best for them. Ideally, all data sources should be made available in multiple formats for ease of use.

### Limited and ambiguous documentation

We did identify instances in which crosswalks exist, but integration is constrained due to limited documentation and awareness. FPED was created to integrate FNDDS with the Dietary Guidelines for Americans so that FNDDS foods could be characterized and assessed based on food patterns used in the Dietary Guidelines for Americans recommendations [38]. Unfortunately, there is no mention of FPED in FNDDS documentation. As a result, users of FNDDS may not know that FPED exists. Additionally, SR Legacy can be integrated with the USDA Special Interest Databases on flavonoids using the nutrient database number, yet this is mentioned in only the Special Interest Databases documentation code book [39]. Solving this problem can be as simple as informing users of crosswalks and best practices.

## Conclusion and Perspectives

Data from multiple domains must be joined into a common analytical framework to facilitate interoperability—this is the most critical action needed to support comprehensive diet sustainability analysis.

The present work proposes the use of food data systems visualizations as a highly valuable tool in strategizing the most effective path forward. In addition, technologic progress will offer opportunities for big data in nutrition to be centrally housed so that accessibility, metadata, formatting, and documentation will no longer be barriers to research. Closing the existing data gaps (or “data canyons” [11]) will require the collaboration of both public and private entities and prolonged efforts by interested stakeholders.

Increasing the interoperability of food systems data will require applying standards for metadata such as FAIR principles, providing clear and expansive data documentation detailing the connections between data sources, and developing standardized ontologies for use in transdisciplinary research.

A common analytical framework would facilitate studies on affordable nutrient density, the environmental aspects of food production, and the likely impact of food systems on climate change [5]. Such transdisciplinary analyses inform public policy, both domestic and international, on food systems transformation and sustainable healthy diets.

## Authors contributions

The authors’ responsibilities were as follows – AD, EJD, SF: conceived the project and developed the overall research plan; EJD, AD: designed the research data analysis plan; EJD: performed analysis; EJD, AD, SF: wrote the paper; SF, AD: performed study oversight; all authors: read and approved the final manuscript.

## Conflict of interest

AD is a member of the Scientific Advisory Boards for Nutrient Institute, Nestlé and FrieslandCampina, the Scientific Cabinet for the National Pork Board, an invited member of the Quality Carbohydrate Coalition’s Scientific Advisory Council (QCC-SAC) funded by Potatoes USA, and has received grants, contracts, and honoraria from entities both public and private with an interest in protein quality, nutrient density metrics, and nutrient profiling of foods. SMF is the Executive Director of and EMJ-D is a contractor of the Nutrient Institute 501(c)(3). Other author reports no conflicts of interest.

## Funding

This work was supported by the Nutrient Institute, a 501(c)(3) not-for-profit organization.

## Data Availability

Data described in the manuscript, code book, and analytic code will be made available upon request.

## Appendix A Supplementary data

Multimedia component1 Multimedia component1

Multimedia component2 Multimedia component2
