# Supplementary figures and images for: Visualizing Data Interoperability for Food Systems Sustainability Research—From Spider Webs to Neural Networks
Source: Curr Dev Nutr. 2023 Sep 29;7(11):102006. doi: 10.1016/j.cdnut.2023.102006 (PMC10616130; doi:10.1016/j.cdnut.2023.102006)

## Supplemental Figure 1

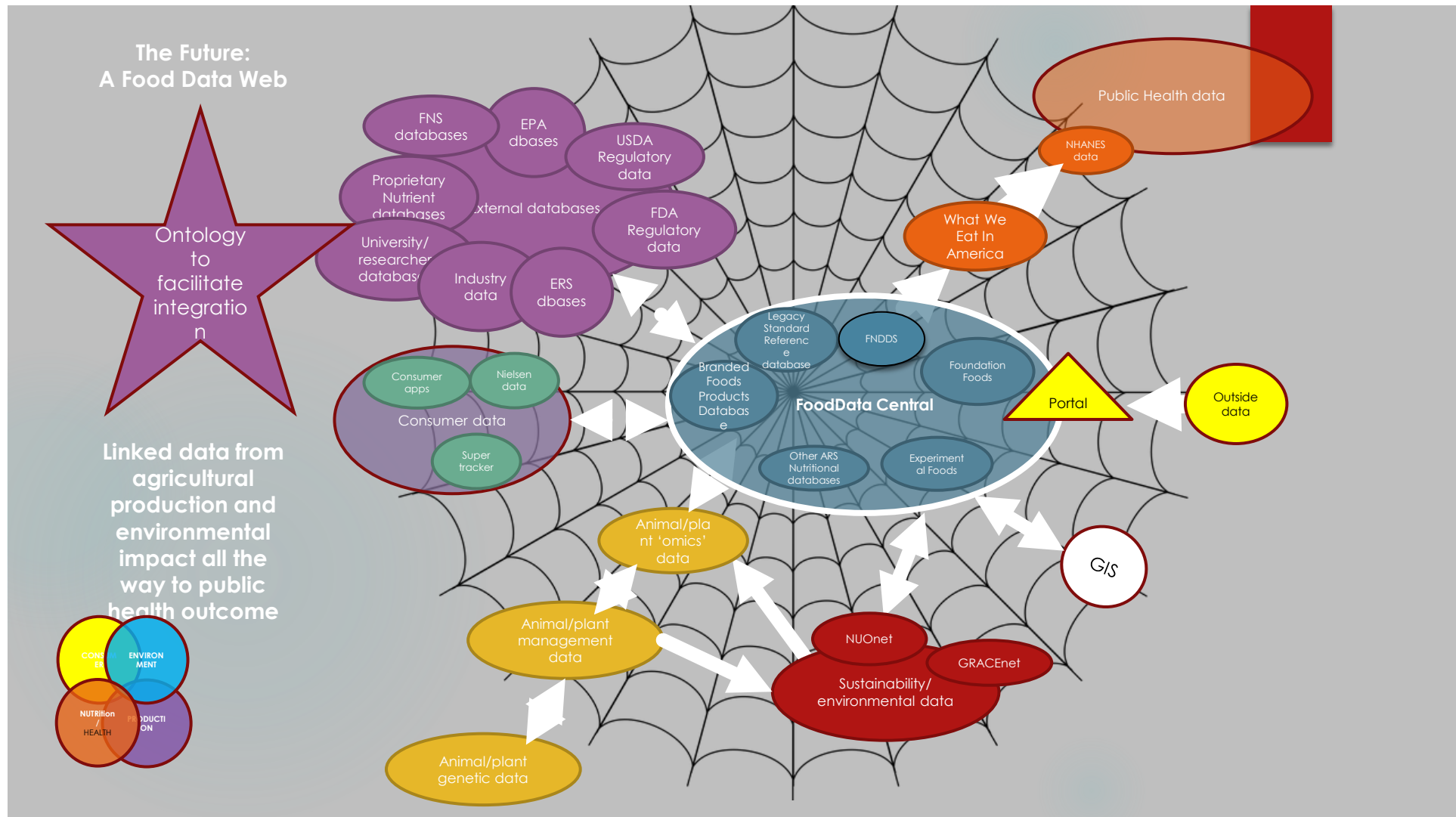

**Footnote:** Courtesy of John Finley USDA (16)

Supplement: Multimedia component1 [file mmc1.pdf]
